# Supplementary figures and images for: TET proteins regulate Drosha expression and impact microRNAs in iNKT cells
Source: Front Immunol. 2024 Sep 19;15:1440044. doi: 10.3389/fimmu.2024.1440044 (PMC11446755; doi:10.3389/fimmu.2024.1440044)

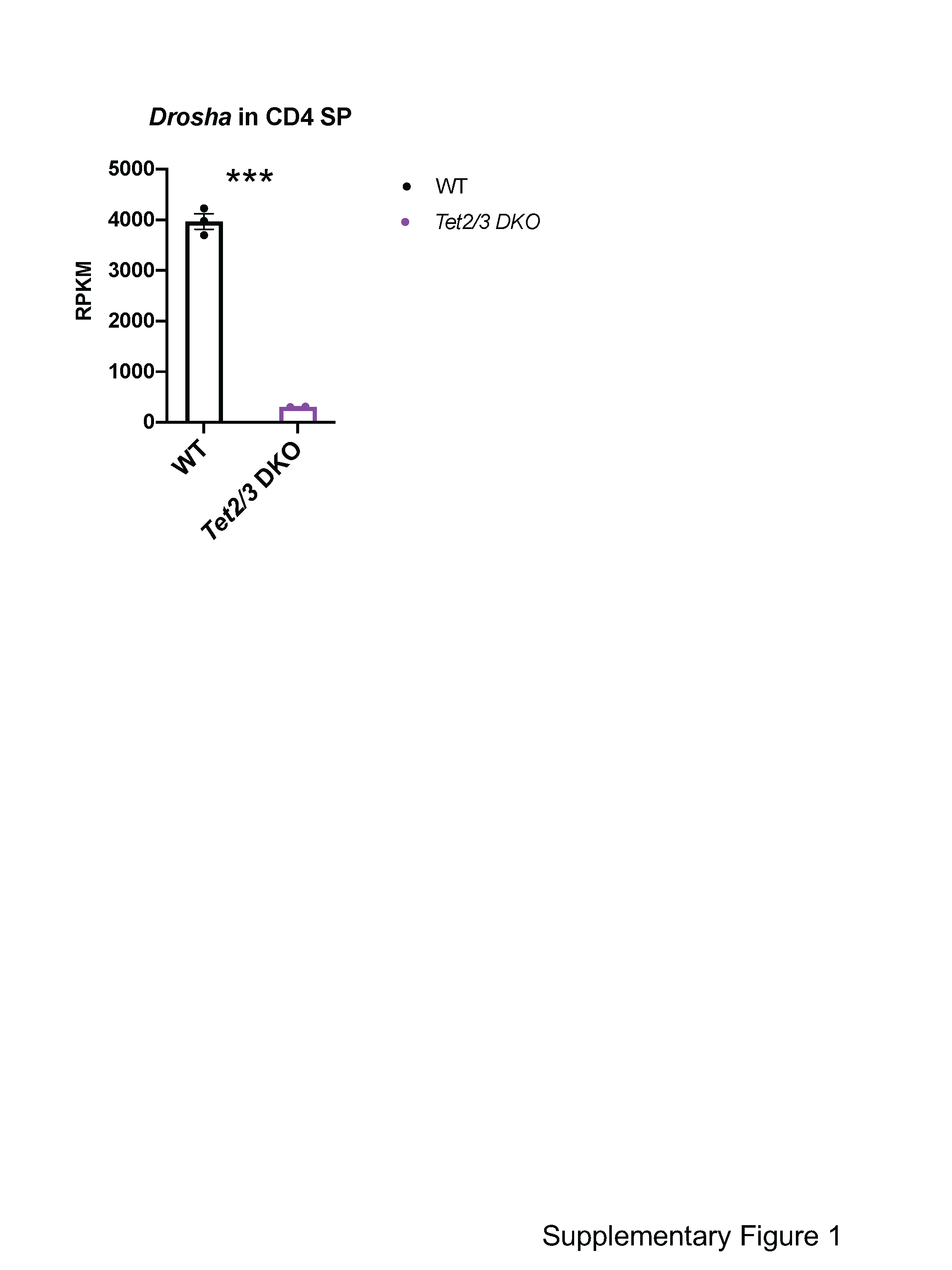

Supplement: Supplementary Figure 1 — TET2 and TET3 regulate expression of Drosha in thymic CD4 SP cells. Gene expression of Drosha in WT (in black) and Tet2/3 DKO thymic iNKT cells (in purple), evaluated by RNA-seq. 3 biological replicates for WT and 2 biological replicates for Tet2/3 DKO CD4 SP cells were assessed. ***(p =0.0003), unpaired t test. Each dot represents an individual biological replicate. Horizontal lines indicate the mean (s.e.m.). [file Image1.tiff]

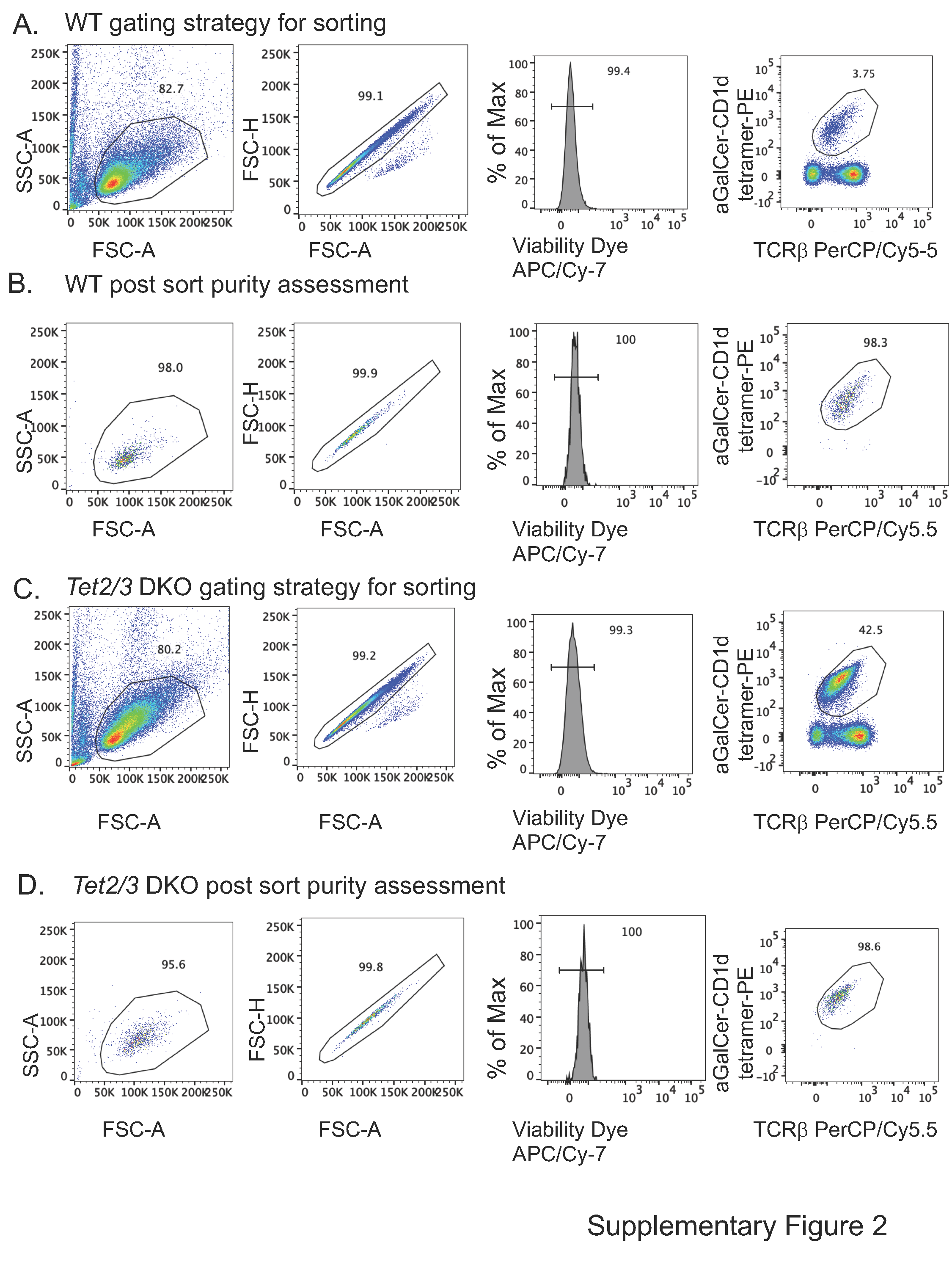

Supplement: Supplementary Figure 2 — Sorting strategy and purity assessment of FACS sorted iNKT cells. (A) Representative flow cytometry plots indicating the gating selection to exclude doublets and isolate live (LD APC/CY7 negative), wild type iNKT cells (aGalCer loaded tetramer positive, TCRβ intermediate cells) by FACS sorting. (B) FACS plots indicating purity of a representative sample of wild type iNKT cells after FACS sorting. (C) As in (A) for Tet2/3 DKO iNKT sample. (D) FACS plots indicating purity of a representative sample of Tet2/3 DKO iNKT cells after FACS sorting. [file Image2.tiff]

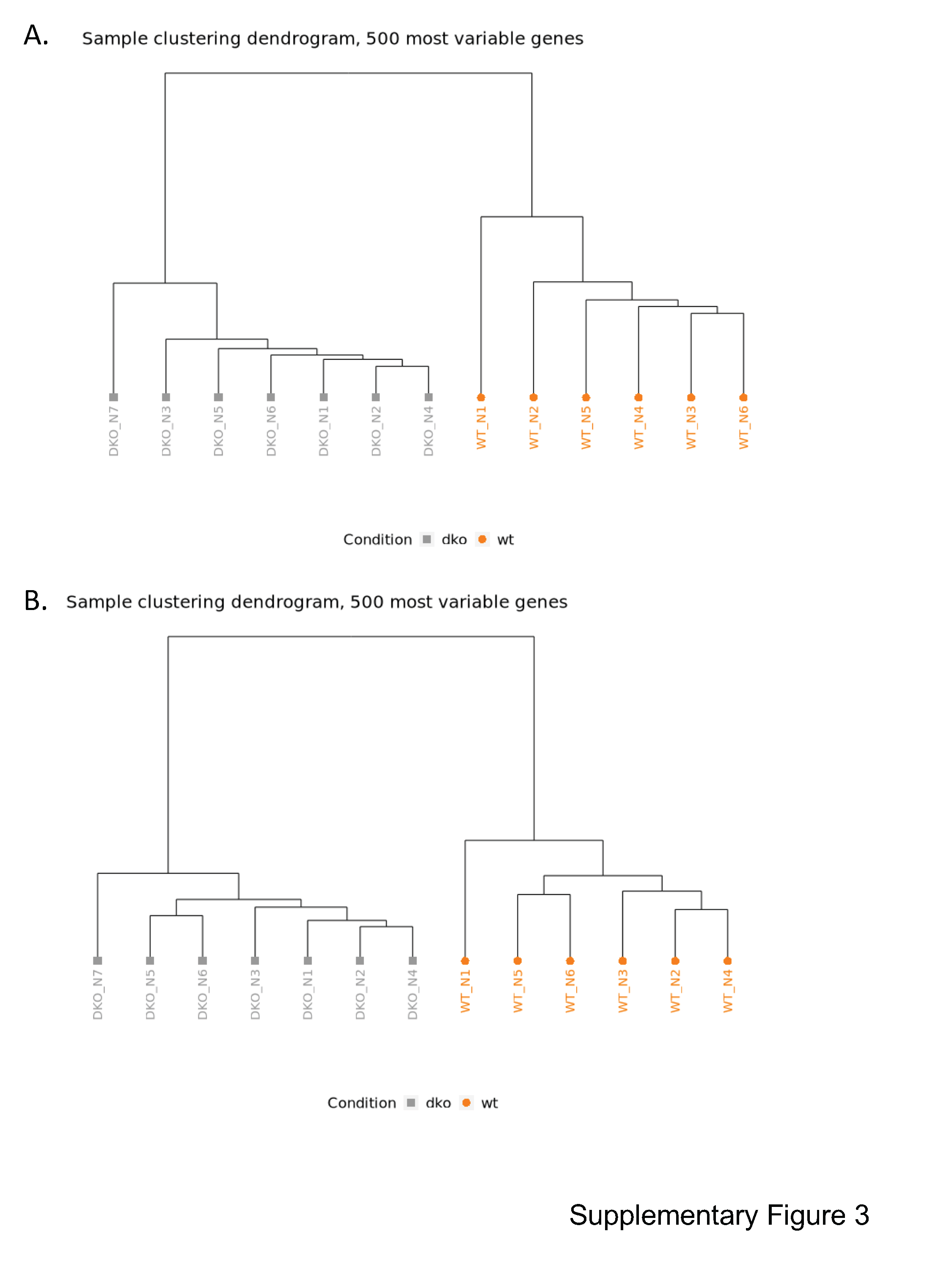

Supplement: Supplementary Figure 3 — Evaluating similarity of RNA samples. (A) Dendrograms indicating the clustering of precursor miRNAs and (B) mature miRNAs samples. [file Image3.tiff]

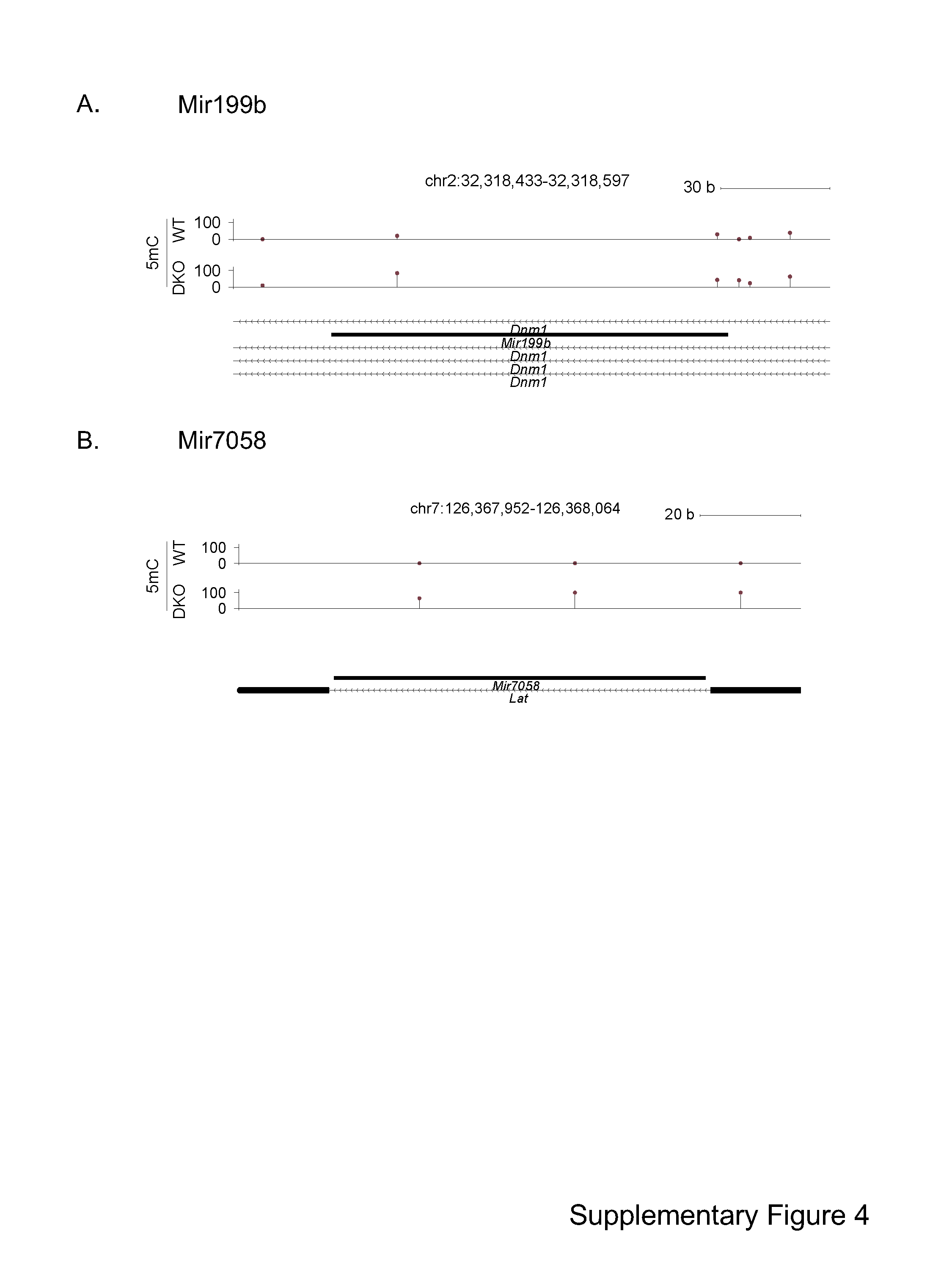

Supplement: Supplementary Figure 4 — Methylation portraits in mature miRNAs. Assessing cytosine methylation by WGBS revealed some gain of methylation in Tet2/3 DKO iNKT cells in two of the mature miRNAs that were downregulated in Tet2/3 DKO iNKT cells. 5mC distribution in WT and Tet2/3 DKO iNKT cells for A. Mir199b and B. Mir7058. [file Image4.tiff]
